# Toxicology

**Published:** 1862-06

**Authors:** Gerard Arink

**Affiliations:** Rochester, N. Y.


Toxicology.—*By Gerard Arink, M. D.. of Rochester, N. Y.*—Poisoning results not only from substances taken into the stomach, but also and still oftener by deadly influences operating in the lungs, or received into the system by means of the skin.

All extraneous matter that becomes mingled with the pure atmosphere, or any decomposition of the same, causes an irrespirable air. This may be shown variously; for instance, suicides are frequently accomplished, especially in France, by the burning of charcoal in a small room, whereby the air becomes vitiated.

Cases of murder are on record, where death has been produced on persons sleeping in contracted dormitories by the closing of all apertures for ventilation. An instance occurs to us of an angry ship's mate who killed his master, with his wife and four children, in this manner, while they were asleep in a small cabin.

In medical practice the use of anæsthetics and medicated vapor inhalation has not unfrequently produced death, or worked with poisonous effects upon the system. A word here in passing in regard to the use of the last named so-called remedial agent. It appears obvious that if anything could be devised that might be efficacious for diseased lungs, it certainly never would reach the desired place by inhalation, for the reason that when the lungs are abnormal, the bronchial tubes are closed up with mucus, just as the nostrils are in the case of cold in the head; in which case the healthy portion of the lungs has to perform the functions of the whole lungs, and in the process of inhaling a medicated vapor designed, but ineffectual, for the *sick* lung, it also becomes weakened and diseased, being robbed of that vital agent, God's pure unmixed air, prepared and fitted so beautifully for the human lungs, and as essential to their proper working as water for the locomotive.

Through ignorance of the simplest, but at the same time a most important law of nature, a process of slow poisoning is continually going on in the case of infants, in which the mothers themselves are the poisoners. The almost idolatry with which many mothers regard their little ones induces them to hang over them and fondle them almost incessantly; holding them face to face, and even at night the baby often lies on the same pillow. Thus in all these ways the exhaled, dead, carbonated air from the lungs of the mother passes over to the child, whose rapidly developing organs require every particle of the purest atmosphere that can gain contact with its little body. In a measure also the breath of the child so incessantly inhaled injures the mother. Thus we often find that the infants of the poor and more industrious classes are healthier and better developed than the children of the rich; for the reason mainly that they are left more to themselves, and so nature has a better chance to do her own work of developing, etc., thoroughly.

All ill ventilated rooms, and rooms where too many live together, oftentimes crowded school-rooms, are fatal to health, and too frequently to life itself. The dry and heated atmosphere existing so generally in houses warmed by a furnace, "that horrible Borgia in a cellar," as it has been aptly styled, is sure in time to undermine the health by its poisonous influences.

Application of poisons upon the skin, or under the epidermis, arrow poisoning, bites and stings of insects, reptiles, animals, etc., can and often do prove fatal.

Having now considered the three different forms of poisoning, by which the deadly agent operates upon the system to destroy its vitality, viz., by the stomach, through the lungs, and by way of the skin, a few remarks on the general mode of treatment to be observed in said cases may be interesting.

The discharging of poisons (venous) from the system, or the preventing of their absorption, can be accomplished in various ways: by pressing; washing; drawing by means of cups; the application of ligatures above the affected parts, like the bandage commonly used in venesection; by cutting out the injured or wounded part; by cauterization, moxa, cauterium actuale et potentiale. If the poison has entered into internal parts, such as the mouth, throat, œsophagus, aspera arteria, lungs, stomach, intestines, bladder, or vagina, the treatment must be such as is best adapted to work upon these different localities. To discharge poisonous matter from the stomach, an emetic or stomach-pump may be used. Emetics are especially efficacious when given very speedily after the poison has been swallowed. The action of an emetic is twofold, as it operates to clear out the stomach of its contents, and produces a certain action of the skin, operating as an excellent diaphoretic. Emetics should in no case be administered where evidences of gastro-enteritis are manifest, or when already symptomatic vomiting exists.

In cases of poisoning by mineral acids or by strong vegetable acids, emetics are not required, but diluted alkalies are demanded; so also when caustic alkalies have been taken, diluted acid is used for neutralizing the same. When the poison taken belongs to the venena acria, such as arsenic and sublimatum, an emetic of pulvis ipecacuanha should be given, one scruple pro dosis, every five minutes, to be repeated three or four times; at the same time should be drank plenty of cold milk and water, white of eggs mixed with water, or sugar and water in good quantity, so as to wash the stomach thoroughly out.

When the poison belongs to the narcotica tetanica, such as strychnine, then stronger working emetics are required; for instance, zinci sulphas, to be given one scruple pro dosis, two or three times, dissolved in water; also cupri sulphas in a powder of five grains, every five minutes, to be repeated two or three times, at the same time drinking freely of cold water, or, better still, cold tea.

When emetics, such as the above named, are not at hand, may be used instead a teaspoonful of snuff, mixed with wine or water, or two teaspoonsful of mustard powder, mixed in warm water.

When at any time it is not practicable to pass anything through the mouth, a syringe may be used with a tube so bent as to pass through the nose into the œsophagus.

Occasion for the use of the stomach-pump has its peculiar indications and contraindications. It is not only used for pumping or sucking out, but also for injecting, washing, dilating and neutralizing. Indications for the use of the stomach-pump, above all other remedies, are when the patient is in a state of insensibility or sopor, unable to swallow anything in short, which is common as the result of narcotic, alcoholic and prussic acid intoxication. When by excessive drinking, especially of beer, the stomach becomes so much distended as to have lost its power of contraction.

We know of a case where a man had thus drunk beer to excess, so that he could not contain another drop, the warmth of his body caused an expansion of the fluid. Emetics were powerless, there was no room for them to work; the stomach had moreover lost its contractibility; inflammation set in, and the man was soon dead. Now, had the stomach-pump been promptly applied in this case, and the stomach discharged of its troublesome burden of fluids, a life might have been saved.

The double stomach-pump of Weis or Charrière may be recommended as being the most simple and easy of application.—*Medical and Surgical Reporter.*

